# Skipping Posterior Dynamic Transpedicular Stabilization for Distant Segment Degenerative Disease

**DOI:** 10.1155/2012/496817

**Published:** 2012-10-03

**Authors:** Bilgehan Solmaz, Ahmet Levent Aydin, Cengiz Gomleksiz, Yaprak Ataker, Mehdi Sasani, Tunc Oktenoglu, Ali Fahir Ozer

**Affiliations:** ^1^Neurosurgery Department, Medistate Hospital, Istanbul, Turkey; ^2^Neurosurgery Department, Istanbul Physical Therapy and Rehabilitation Training Hospital, Istanbul, Turkey; ^3^Neurosurgery Department, School of Medicine, Mengücek Gazi Training and Research Hospital, Erzincan University, Erzincan, Turkey; ^4^Physical Therapy and Rehabilitation Department, American Hospital, Istanbul, Turkey; ^5^Neurosurgery Department, American Hospital, Istanbul, Turkey; ^6^Neurosurgery Department, School of Medicine, Koc University, Istanbul, Turkey

## Abstract

*Objective*. To date, there is still no consensus on the treatment of spinal degenerative disease. Current surgical techniques to manage painful spinal disorders are imperfect. In this paper, we aimed to evaluate the prospective results of posterior transpedicular dynamic stabilization, a novel surgical approach that skips the segments that do not produce pain. This technique has been proven biomechanically and radiologically in spinal degenerative diseases. *Methods*. A prospective study of 18 patients averaging 54.94 years of age with distant spinal segment degenerative disease. Indications consisted of degenerative disc disease (57%), herniated nucleus pulposus (50%), spinal stenosis (14.28%), degenerative spondylolisthesis (14.28%), and foraminal stenosis (7.1%). The Oswestry Low-Back Pain Disability Questionnaire and visual analog scale (VAS) for pain were recorded preoperatively and at the third and twelfth postoperative months. *Results*. Both the Oswestry and VAS scores showed significant improvement postoperatively (*P* < 0.05). We observed complications in one patient who had spinal epidural hematoma. *Conclusion*. We recommend skipping posterior transpedicular dynamic stabilization for surgical treatment of distant segment spinal degenerative disease.

## 1. Introduction

The most frequent clinical problem of the adult spine is back pain. It is known that 60–80% of the population will have back pain at some point in their lives that may affect their general health, daily activities, and their working capacity. It is assumed that back pain only has a defined pathology in 15% of patients [1], and dysfunctional segmental motion and discogenic pain are problems that may need to be treated surgically. According to Bertagnoli, disc-related spinal problems could be treated because of the state of degenerative segmental alterations [2]. Those at an earlier stage of disc degeneration may respond to the conservative treatment. More advanced disc degeneration may require open-disc surgery, especially concomitant nerve root compression. Fusion surgery is usually indicated in more advanced segmental degeneration. Discectomy and fusion are performed with the aim of reducing pain and eliminating neural compression rather than restoring disc or segmental function. Researchers have demonstrated the benefits of fusion over nonsurgical treatment in the alleviation of chronic low-back pain [3, 4]. Although studies have shown improvements in the instrumentation techniques that have increased the radiological fusion rate to >94%, they have failed to provide evidence of actual improvements in clinical outcome [5].

Retrospective clinical studies have demonstrated that the lumbar fusion can lead to an acceleration in pathologic changes of the adjacent motion segment [6]. The quest for a more physiological surgical solution than fusion has initiated the so-called nonfusion technologies. One of them, posterior dynamic stabilizing implants, is intended to realign and stabilize one or more linked vertebral segments without complete immobilization of the segments. Therefore, we planned a novel surgical approach using posterior dynamic transpedicular stabilization (PDTS) to relieve pain and reduce morbidity and mortality. This paper presents prospective results of skipping the segments that do not cause pain. This technique is proven biomechanically and radiologically as a surgical approach of PDTS in spinal degenerative diseases.

## 2. Materials and Methods

**Figure 1:**
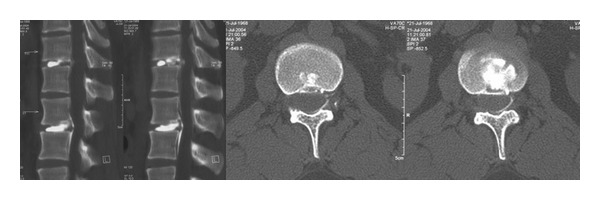
Provocative discography shows degeneration of the disc.

**Figure 2:**
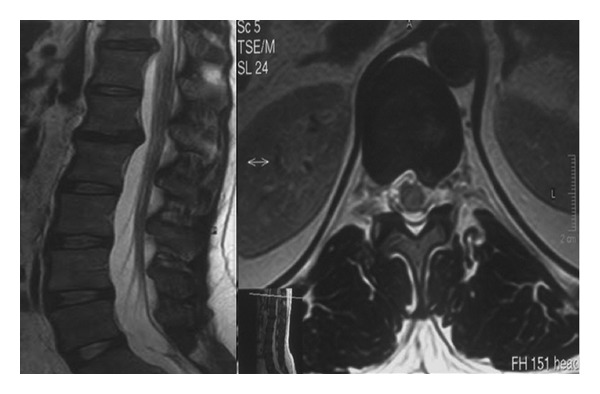
The patient has two distinct degenerative disc diseases in the spine. The rest of the vertebral column has no problem.

**Figure 3:**
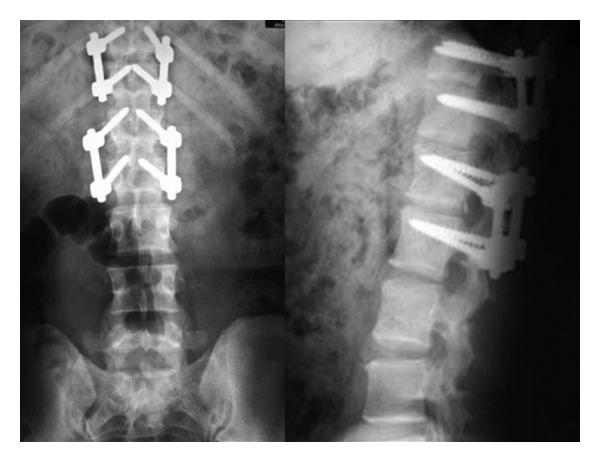
Two different levels of posterior dynamic stabilizations were performed in the same patient.

This study included 18 patients averaging 54.94 years of age; there were 10 females and 8 males. Our selection criteria for this procedure included any neurogenic, radicular pain and/or chronic low-back pain that was resistant to any conservative treatment and neurological deficit. The level of provocative pain was determined by discography in cases where the source of pain was not confirmed by clinical and radiological findings (Figure 1). Radiological evaluations prior to magnetic resonance (MR) (Figure 2) and after surgery consisted of anteroposterior (AP) and lateral X-ray studies (Figure 3). Primary indications, demographic data, and details of the operations performed are shown in Table 1.

**Statistical Methods**

The Oswestry Disability Index (ODI) and the visual analog scale (VAS) were used for preoperative and postoperative subjective patient evaluations.

Preoperative and postoperative values (VAS and ODI) were compared with the Wilcoxon ranked sum test. 

## 3. Results

Oswestry scale and VAS scale values were compared between the following groups: preoperative and 3-month postoperative, preoperative and 12-month postoperative, 3-month postoperative and 12-month postoperative. We observed significant changes between the groups. The preoperative mean Oswestry scale score was 68.00, the 3-month postoperative mean Oswestry scale score was 23.89, and the 12-month postoperative mean Oswestry scale score was 14.00. This decrease in the mean Oswestry scale score as the postoperative time increased was significant (*P* < 0.05, Wilcoxon test). The preoperative mean VAS score was 7.28, the 3-month postoperative mean VAS score was 2.50, and the 12-month postoperative mean VAS score was 1.33 (Table 2). This decrease in the mean VAS as the postoperative time increased was also significant (*P* < 0.05, Wilcoxon test).

## 4. Discussion

Back pain at a symptomatic motion segment may originate from vertebral endplates, disc annulus, vertebral periosteum, facet joints, and/or surrounding soft tissue structures [7]. These structures also contribute to the biomechanical stability of the spinal column. The pathology of discogenic pain and degenerative instability has been described by Kirkaldy-Willis and Farfan [8]. Pain is reported to be the simplest description as well as the major symptom of instability. If a patient's limited instability is the result of glacial instability and dysfunctional segmental motion, he or she will experience pain but will be able to lead a life without neurologic deficit [9]. Recent studies have suggested that the chronic instability related to the disc or vertebral body degenerative changes associated with abnormal motion results in the potential for pain. 

The management of painful, unstable spinal segments due to degenerative disorders is much more difficult than spinal trauma or tumor surgery. During spinal surgery, instability must be taken into account when relieving pain with adequate neural tissue decompression. Whenever possible, bone, joint, ligament, and muscle tissues must be preserved, and suitable fixation methods must be chosen for the damaged ones; otherwise, decompressive procedures may induce or increase instability [10–13]. 

The concept of spinal segment arthrodesis as a treatment for unstable degenerate spinal segments evolved almost 100 years ago. Spinal fusion was first described by Albee [3], for the treatment of Pott disease, and Hibbs [14], who performed spinal fusion as a treatment for spinal deformity. Since that time, fusion has been the conventional surgical treatment for chronic low-back pain attributed to degenerative disorders. For a long time, fusion was thought to be a necessity for a successful outcome, but the results of many recent studies have challenged this concept by showing that patients had limited improvements in pain relief and increased mobility after surgery [15–18]. Posterior spinal instrumentation and fusion led to deteriorated biomechanical properties of the ligamentum flavum, posterior longitudinal ligament, and interspinous and supraspinous ligaments [19]. Adequate preservation of the partial lamina, spinous process, and supraspinous and interspinous ligaments during laminectomy was helpful for alleviating the stress on the adjacent segment. Furthermore, biomechanical studies showed that posterolateral fusion with hemilaminectomy had less stress concentrated on the adjacent disc than posterolateral fusion with total laminectomy had on flexion [20]. Moreover, fusion eliminates the motion of the functional spinal segment and may overload the adjacent segments. Indeed, adjacent segment degeneration is a known consequence of spinal fusion [3, 4, 21–25]. Lehmann et al. reported accelerated degenerative changes of the adjacent segment and segmental instability above the fusion in 45% of their patients [26]. Major drawbacks of spinal fusion are stiffness, pseudoarthrosis, mechanical failure, and/or adjacent degenerative disease [27–31]. Because of these problems, less rigid stabilization systems have recently become more popular in spine surgery. Mobile stabilization systems have been shown to neutralize injurious forces and restore normal functions of the spine segments while also protecting the adjacent segments [32]. One of the motion preservation technologies is the dynamic pedicular screw-rod system. It is a nonfusion dynamic implant system that controls displacement in rotation and translation as well as providing stabilization. This system allows potential sagittal mobility at the hinge site between the screw head and the shank of the screw. Mobility occurs mechanically between the longitudinally oriented rod and the sagittally placed screw shank. This articulated connection between the rod and the screw caused a reduction in flexion strain and resulted in a lower rate of implant failure [33]. In these systems, the stress load was transferred from the implant to the spine, which decreased the tension on the bone [34]. Although it tends to restrict mobility in flexion, extension, lateral bending, and axial rotation, a sharing of the motion and stress load still permits movement, prevents deterioration of the neighboring superior disc level, and slows the process of degenerative progression at the adjacent levels [35]. Further, this controlled distribution of load between the implant and the spine may reduce postoperative damage to joint segments. 

Microdiscectomy using Yasargil's microscope has been the standard surgical treatment for symptomatic disc herniation. However, treatment of disc herniations with degenerative changes is still a matter of debate. A simple discectomy cannot stop the continuing segmental degeneration and in some instances can even cause an acceleration of this process [6, 36–39]. In addition, the segmental fusion leads to an irreversible loss of function of the treated segment and a resulting risk of adjacent segmental degeneration and pseudoarthrosis [6, 36–39]. Disappointing surgical outcomes observed in the literature were generally due to increasing instability after surgery [40, 41]. Additional dynamic stabilization, applied by Putzier et al., showed significantly fewer signs of progressive degeneration and stabilized the motion segment after nucleotomy to prevent further disc degeneration [35]. Kaner et al. showed the importance of the annular defect. They concluded that to apply a posterior dynamic stabilization is reasonable to the patients with disc herniation and an annular defect larger than 6 mm [42]. 

In spinal stenosis with degenerative spondylolisthesis, decompression and fusion are widely recommended. Clinical improvements observed in patients with spinal stenosis and degenerative spondylolisthesis generally depend on the effectiveness of the neural decompression; however, the value of instrumentation is still a matter of debate. Some surgeons recommend fusion with instrumentation because it prevents further sliding and applies more decompression without the risk of destabilization. Studies show that the decompression combined with arthrodesis (posterolateral or interbody) significantly improved patient outcome compared to decompression alone [43–48]. However, some studies revealed that the additive instrumentation leads to higher fusion rates and less progression of spondylolisthesis; however, it is unlikely to improve clinical outcome [17, 46, 49–52].

Surgeons advocating fusion without instrumentation indicate that this process gives a little chance to adjacent segment degeneration, and this concept is supported by various studies. Fischgrund et al. reported a randomized study comparing decompressive laminectomy and arthrodesis with or without spinal instrumentation. After a 2-year followup period, the authors concluded that successful fusion did not influence the patients' outcomes [17]. Considering the older age of patients, major lengthy procedures like a fusion operation, especially posterolateral and interbody fusion with autogenous iliac crest graft, are associated with wound problems in the donor site, neurovascular damage, infections, pelvic fracture, and bleeding, all of which increase morbidity and mortality [53, 54]. Because the degenerative spondylolisthesis is a dynamic process, the purpose of fusion should be to prevent listhesis rather than cure the mechanical pain. Recent studies suggest that the dynamic stabilization system may maintain enough stability to prevent further translation of vertebra without fusion [55–57]. 

Lumbar arthrodesis for the management of an unstable spine has dramatically increased over the last few decades. The purpose of spinal instrumentation is to increase fusion, but it is obvious that fusion with very long segment instrumentation is being tried, especially for different segment degenerative disorders. 

In our study, we adopted a surgical approach that only targeted unstable degenerated and pain-producing segments. Using this approach, we applied neural decompression by a minimally invasive approach that resulted in very little disruption of tissue integrity, discectomy by sparing ligamentum flavum, and decompression by hemilaminectomy in stenosis [58]. We provided stabilization of pain-producing degenerated unstable segments by posterior dynamic transpedicular instrumentation while skipping stable segments. Postoperative VAS and Oswestry scores of a limited series of patients treated with this preservative surgical approach were satisfactory. 

## Figures and Tables

**Table 1:** Patient demographic data.

Patient No.	Gender	Age	Preoperative neurological findings	Radiologic findings (X-Ray, MRI)	Operation	Complication
1	M	66	Bilateral SLR 30°+ Left FST+ Left gastrocnemius 2/5 MW	Left L3-4 HNP Right L5-S1 HNP L3-4 spinal stenosis	Left L3-4, right L5-S1 microlumbar discectomy L3-4, L5-S1 PDI	None

2	F	60	LBP	L3-4, L4-5 DDD	L3-4, L4-5 PDI	None

3	F	48	LBP Left FST+ Left SLR 60°+	Left L3-4 HNP Right L5-S1 HNP	Left L3-4, right L5-S1 microlomber discectomy L3-4, L5-S1 PDI	None

4	F	74	LBP Left L5 dermatomal hypoesthesia	L1-2, L4-5 spinal stenosis	L1, L4 hemilaminectomy L1-2, L4-5 PDI	None

5	F	60	LBP Left SLR 60°+	L1-2 midline HNP L5-S1 left HNP	Right L1-2, left L5-S1 microlumbar discectomy + L1-2 and L5-S1 PDI	None

6	M	40	LBP	T11-T12, L1-2 DDD	T11-T12, L1-2 PDI	None

7	F	54	LBP	L2-3, L4-5, DDD, spondylolisthesis	L2-3, L4-5 PDI	None

8	M	53	LBP	L5-S1 and L1-2, L2-3 DDD	L1-2, L2-3, L5-S1 PDI	None

9	M	47	LBP Right SLR 45°+ S1 dermatomal hypoesthesia	L3-4 DDD right L5-S1 HNP	Right L5-S1 microlomber discectomy L3-4, L5-S1 PDI	None

10	M	29	LBP	L3-4, L5-S1 DDD	L3-4, L5-S1 PDI	None

11	F	49	Left FST+ Left SLR 30°+ Left achilles reflex hypoactive Left gastrocnemius L5-S1 1/5 MW	Left T12-L1 HNP Left L5-S1 HNP L4-5 retrolisthesis	Left T12-L1, L5-S1 microlomber discectomy L4-5-S1 PDI	None

12	F	74	LBP	L3-4, L5-S1 DDD	L3-4, L5-S1 PDI	Spinal epidural hematoma

13	M	50	LBP Left SLR 45° Left L4 dermatomal paresthesia	Left L3-4, L5-S1 HNP Foraminal stenosis	Left L3-4, L5-S1 microlomber discectomy L3-4, L5-S1 PDI	None

14	F	48	LBP	L1-2 HNP, L1-2, L4-5, L5-S1 DDD	Left L1-2 microlomber discectomy Left L4-5 microlomber decompression, L1-2, L4-5-S1 PDI	None

15	M	73	LBP	L5-S1 DDD T12-L2 foraminal stenosis	L5-S1, microlomber discectomy L5-S1, T12-L2 PDI	None

16	F	68	LBP	L2-3, L5-S1 DDD	L2-3, L5-S1 PDI	None

17	F	54	LBP	L1–3 and L4-5 DDD	L1–3 and L4-5 PDI	None

18	M	42	LBP	L2-3 and L4-5 DDD	L2-3 and L4-5 PDI	None

PDI: posterior dynamic instrumentation, FST: femoral stretching test, SLR: straight leg rising, HNP: hernia nucleus pulposus, LBP: lower back pain, and DDD: degenerative disc disease.

**Table 2:** Patient preoperative and postoperative data.

Patient no.	Preoperative VAS	Postoperative 3-month VAS	Postoperative 12-month VAS	Preoperative Oswestry	Postoperative 3-month Oswestry	Postoperative 12-month Oswestry
1	5	3	1	56	48	10
2	8	2	2	58	32	24
3	6	3	2	68	32	24
4	7	3	1	52	24	6
5	7	3	2	72	28	16
6	6	4	2	56	32	26
7	7	2	1	74	24	16
8	8	3	2	68	48	26
9	7	1	1	64	8	12
10	8	2	2	78	6	12
11	8	3	2	64	18	16
12	8	2	2	72	12	8
13	7	3	3	68	24	18
14	6	3	2	64	18	12
15	8	2	1	78	18	12
16	9	3	0	74	24	6
17	8	1	1	82	18	8
18	8	2	0	76	16	4

Mean	7.28	2.50	1.33	68.00	23.89	14.00
